# Clinical outcomes and risk factors in pediatric patients with solitary functioning kidney: a comparative analysis of congenital and acquired etiologies

**DOI:** 10.3389/fped.2025.1574000

**Published:** 2025-04-24

**Authors:** Hülya Gözde Önal, Yonca Semet

**Affiliations:** ^1^ Department of Pediatric Nephrology, Samsun Training and Research Hospital, Samsun, Türkiye; ^2^ Department of Pediatric Nephrology, Faculty of Medicine, Samsun University, Samsun, Türkiye

**Keywords:** solitary functioning kidney, renal agenesis, multicystic dysplastic kidney, pediatric nephrology, nephrectomy

## Abstract

**Introduction:**

This study aims to assess the clinical outcomes and kidney function in pediatric patients with a solitary functioning kidney (SFK) due to various causes. It focuses on evaluating the glomerular filtration rate (GFR) across five groups: renal agenesis, multicystic dysplastic kidney (MCDK), atrophic kidney, hypoplastic kidney, and patients who underwent nephrectomy due to bleeding, stones, infection, or tumor without having progressed to renal failure.

**Methods:**

A retrospective chart review was conducted on single kidneys of 396 patients seen at the pediatric nephrology clinic from January 2011 to June 2024. Descriptive statistics were used to summarize the data, with chi-square tests employed for categorical variables and *t*-tests or Mann–Whitney *U*-tests used for continuous variables. For comparisons involving more than two groups, ANOVA or Kruskal–Wallis tests were performed, followed by *post-hoc* Bonferroni tests.

**Results:**

Baseline and final GFR values showed significant differences between the groups in the pre- and post-tests. The MCDK group had significantly lower GFR than the renal agenesis group and the remaining etiologies. Albumin levels were decreased in MCDK patients, whereas neutrophil levels were elevated in patients with renal agenesis. Hypertension or proteinuric activity did not exhibit significant variations in the frequency across these groups.

**Discussion:**

This study highlights the importance of a personalized management approach for pediatric patients with an SFK, specifically those with MCDK, who are more likely experience early loss of kidney function. Minimizing the risks and improving the outcomes would require a routine, topical course of monitoring, along with appropriate treatment.

## 1 Introduction

A solitary functioning kidney (SFK) describes a condition in which a single kidney performs all renal functions due to the other kidney being non-functional or absent. SFK has been known to result from developmental defects such as renal agenesis and multicystic dysplastic kidney (MCDK) or from acquired conditions such as surgical removal of a kidney (done after trauma or infections). In SFK cases, compensatory structural changes can be seen, which include hypertrophy and hyperplasia. Hypertrophy refers to an increase in the size of existing nephrons, while hyperplasia involves the formation of additional nephrons during the prenatal period as a compensatory mechanism for nephron loss (1). Studies suggest that during early life, kidney growth exceeding 2 standard deviations (>2 SDS) may reflect an increased number of nephrons formed prenatally as an adaptive response to nephron reduction. These compensatory changes, while initially beneficial, can predispose the kidney to glomerular hyperfiltration, which may lead to systemic hypertension, proteinuria, or chronic kidney disease (CKD) over time (1, 2).

Renal agenesis is a congenital anomaly in which one kidney fails to develop during embryonic development, which is often observed in congenital renal conditions. The condition may manifest without or alongside other urinary, cardiovascular, and genitourinary malformations. Genetic factors, including mutations in the PAX2 and HNF1B transcription factors, have been identified as causes of this condition (3). It has been established that those suffering from renal agenesis are more susceptible to developing CKD; thus, it is appropriate to avoid or limit factors that would place additional burden on the kidney, such as high blood pressure and proteinuria, and ensure follow-up with prenatal ultrasound (USG) from the point of making the diagnosis (4, 5).

Another common congenital defect is MCDK, which results in a developmental abnormality affecting one or both kidneys, leading to cystic changes and loss of renal function. The majority of cases go undiagnosed due to the lack of visible symptoms and are diagnosed during prenatal ultrasound scanning. Although the dysplastic kidney usually resorbs itself over time, MCDK patients remain at risk for hypertension, urinary tract infections (URIs), and obstructive uropathy. This presents a challenge as the preservation of functional renal tissue and the establishment of long-term follow-up care must be devised to prevent CKD and renal scarring (6, 7).

Acquired SFK is also seen in individuals who have undergone a single nephrectomy due to infection, tumor, trauma, or kidney stones. Congenital SFK tends to be characterized by a different age of onset and the amount of stress experienced by the remaining kidney. A single kidney working harder is at high risk for declining GFR and subsequent renal failure. Therefore, it becomes vital to consistently and promptly follow the patient to identify and treat the complications that would expedite CKD progression (8).

Pediatric patients with various causes of SFK, such as renal agenesis, MCDK, atrophic kidney, hypoplastic kidney, and nephrectomy, represent a unique population with distinct clinical outcomes and renal function profiles. The focus of this paper is to evaluate the GFR, identify risk factors such as hypertension and proteinuria, and differentiate based on the biochemical parameters such as albumin, C-reactive protein, and neutrophil count to understand the disease better from the perspective of its evolution and management.

Patients with a single functional kidney tend to develop kidney damage in the long run, and this could be caused by the compensatory hypertrophy of the single reduced functional kidney, eventually leading to CKD. Distinct outcomes, such as compensatory mechanisms based on the etiology of the SFK, can be highlighted. These findings could help develop individualized follow-up and management protocols. This research addresses a significant gap in the literature by examining changes in GFR and other laboratory parameters indicative of renal injury in patients with different SFK etiologies.

The research expands the understanding of chronic risks related to SFK and offers insights into general approaches to CKD management. The work highlights the importance of individually tailored follow-up and multidisciplinary management protocols for patients with SFK by presenting outcome data of different etiologies of SFK. Furthermore, it emphasizes the role of monitoring GFR and its decline and laboratory tests in optimizing patient outcomes and enhancing early intervention strategies.

## 2 Methods

This retrospective study examined 396 SFKs presented at the Pediatric Nephrology Clinic of Samsun Training and Research Hospital from January 2011 to June 2024. Ethical approval was granted by the Non-Interventional Clinical Research Ethics Committee of Samsun University (GOKAEK 2024/18/3), and all operations were conducted in accordance with the ethical principles outlined in the Declaration of Helsinki.

Renal lengths and percentiles were determined using USG based on normative pediatric data. Diagnoses of SFK conditions such as renal agenesis, MCDK, atrophic kidney, hypoplastic kidney, and nephrectomy and its variants were mainly established through USG. In situations where routine static renal scintigraphy [dimercaptosuccinic acid scan (DMSA)] was employed, it was carried out to support the diagnostic processes. When the need arose to distinguish between renal agenesis or atrophic kidney, USG findings were used, supplemented by DMSA imaging when available.

Congenital or acquired conditions characterized above by severely underdeveloped kidney size and function were considered atrophic. Another renal condition was the hypoplastic kidney, which was also poorly developed; the inclusion of hypoplastic kidneys is important as they contribute to the increased functional burden on the opposite kidney, further overloading the solitary functioning kidney, which indicates that it is just one of several contributing factors.

The diagnosis of MCDK was mainly made during the prenatal USG. However, postnatal cases that were reasonably not imaged within the early period of life were occasionally misclassified as renal agenesis. This highlights the significance of the age at diagnosis and the need for conscious long-term review to ensure accurate classification of SFK etiologies.

GFR was estimated using the Schwartz equation by taking into account serum creatinine, height, and concentration of muscle mass. Proteinuria was evaluated by utilizing the spot urine protein-to-creatinine ratio, considering pediatric normative data. The follow-up duration and the age of patients at their last visits were recorded, and prenatal diagnostic information was included when available. Not all pregnancies included antenatal imaging or follow-up, especially in cases where patients did not get prenatal care.

The design of this study allows for a broad and thorough investigation, at least, of SFK, its various causes, and even clinical outcomes. All these processes—accurate diagnosis, adequate follow-up, and targeted therapy—are crucial to achieving satisfactory outcomes in this study.

### 2.1 Statistical analysis

Statistical analyses were conducted using SPSS for Windows (Version 29, Chicago, IL, USA). Categorical variables were presented as frequencies and percentages, while continuous variables were summarized as mean ± standard deviation for normally distributed data or median (IQR) for non-normally distributed data. Normality was assessed using the Shapiro–Wilk test and histograms.

Group differences for categorical variables were analyzed using Pearson's chi-square test or Fisher's exact test, as appropriate, with Bonferroni correction applied for multiple comparisons. For continuous variables, the Student *t*-test was used for normally distributed data, and the Mann–Whitney *U*-test was used for non-normally distributed data. For comparing more than two groups, ANOVA with Tukey's *post-hoc* test was applied to parametric data, and the Kruskal–Wallis test with Bonferroni-adjusted *post-hoc* comparisons was used for non-parametric data. All tests were two-sided, and statistical significance was set at *p* < 0.05.

## 3 Results

A total of 396 patients were included in the study and categorized by etiology into the following groups: renal agenesis (39.9%, *n* = 158), multicystic dysplastic kidney (9.3%, *n* = 37), nephrectomy (6.8%, *n* = 27), atrophic kidney (13.6%, *n* = 54), and hypoplastic kidney (30.3%, *n* = 120). There were no statistically significant differences in the median age or sex distribution across the groups (*p* = 0.238 and 0.607, respectively) (Table 1). However, a significant difference was found in the median follow-up duration between the groups (*p* < 0.001). In the *post-hoc* analysis, the atrophic kidney group [4 (IQR 2–6) years] had a significantly shorter follow-up duration compared to the nephrectomy [7 (IQR 5–9) years, *p* = 0.007], hypoplastic kidney [6 (IQR 3–9) years, *p* = 0.004], and multicystic dysplastic kidney [8 (IQR 4–9.5) years, *p* < 0.001] groups. The follow-up duration in the multicystic dysplastic kidney group was also significantly longer than in the renal agenesis group (*p* = 0.026). All other comparisons between groups were not statistically significant (adjusted *p* > 0.05 for all comparisons).

**Table 1:** Baseline characteristics and follow-up data of patients by etiology.

Characteristics	Renal agenesis (*n* = 158)	Multicystic dysplastic kidney (*n* = 37)	Nephrectomy (*n* = 27)	Atrophic kidney (*n* = 54)	Hypoplastic kidney (*n* = 120)	*p*
Age (years)	12 (7.8–16)	12 (8–16)	14 (9–17)	12 (7–17.3)	13 (9–17)	0.238
Sex						
Male patients	98 (42.2%)	24 (10.3%)	16 (6.9%)	30 (12.9%)	64 (27.6%)	0.607
Female patients	60 (36.8%)	13 (8%)	11 (6.7%)	24 (14.7%)	55 (33.7%)	
Follow-up duration (years)	5 (2–8)	8 (4–9.5)	7 (5–9)	4 (2–6)	6 (3–9)	<0.001
Initial GFR (ml/min/1.73 m^2^)	103 (69.5–125)	68 (58–80)	88 (58–120)	102 (74–124)	90 (78–107.5)	<0.001
Final GFR (ml/min/1.73 m^2^)	98 (78.8–115)	91 (80–102.5)	87 (76–105)	92 (83–103.3)	75 (66–91)	<0.001
Patients with GFR decline	91 (57.6%)	23 (24.3%)	17 (63%)	34 (63%)	78 (65%)	<0.001
Annual GFR^a^ decline rate (ml/min/1.73 m^2^/year)	5.1 (2.7–12)	2.8 (1.9–5.8)	2.4 (1.3–8.1)	6.3 (3.6–11.6)	4.2 (1.8–7)	<0.001
Hypertension	12 (7.6%)	6 (16.2%)	3 (11.1%)	2 (4.1%)	7 (3.7%)	0.192
Proteinuria at first visit	17 (10.8%)	7 (18.9%)	1 (3.7%)	6 (11.1%)	18 (15%)	0.336
Proteinuria at last visit	11 (7%)	4 (10.8%)	1 (3.7%)	4 (7.4%)	6 (5%)	0.730

GFR, glomerular filtration rate.

^a^ The annual GFR decline rate was calculated only for patients who experienced a decline in GFR.

A significant difference in median baseline GFR was observed across the groups (*p* < 0.001). *Post-hoc* analyses showed that the multicystic dysplastic kidney group [68 (IQR 58–80) ml/min/1.73 m^2^] had a significantly lower GFR compared to the atrophic kidney [102 (IQR 74–124) ml/min/1.73 m^2^, *p* < 0.001], hypoplastic kidney [90 (IQR 78–107.5) ml/min/1.73 m^2^, *p* = 0.02], and renal agenesis [103 (IQR 69.5–125) ml/min/1.73 m^2^, *p* < 0.001] groups. No other group comparisons showed significant differences (adjusted *p* > 0.05).

A significant difference in median GFR at the last visit was also noted between the groups (*p* < 0.001). *Post-hoc* analyses showed that the hypoplastic kidney group [75 (IQR 66–91) ml/min/1.73 m^2^] had significantly lower GFR compared to the renal agenesis [98 (IQR 78.8–115) ml/min/1.73 m^2^, *p* < 0.001] and atrophic kidney [92 (IQR 83–103.3) ml/min/1.73 m^2^, *p* < 0.001] groups. No other group comparisons reached statistical significance (adjusted *p* > 0.05).

The proportion of patients with a decrease in GFR differed significantly between the groups (*p* < 0.001). *Post-hoc* analyses revealed that the multicystic dysplastic kidney group had a significantly lower proportion of patients with decreased GFR (24.3%, *n* = 23) compared to the renal agenesis (57.6%, *n* = 91, *p* < 0.001), nephrectomy (63%, *n* = 17, *p* < 0.001), atrophic kidney (63%, *n* = 34, *p* < 0.001), and hypoplastic kidney (65%, *n* = 78, *p* < 0.001) groups. No other group comparisons were statistically significant (adjusted *p* > 0.05).

There was a significant difference in the annual rate of GFR decline between the groups (*p* < 0.001); however, the *post-hoc* analysis lacked sufficient power to identify the specific group responsible for the difference.

No statistically significant differences were observed between the groups in terms of the prevalence of hypertension, the presence of proteinuria at the initial visit, or the presence of proteinuria at the last visit (*p* = 0.192, 0.336, and 0.730, respectively).

As shown in Table 2, there were no significant differences between the groups in terms of median C-reactive protein (CRP), CRP/albumin ratio, white blood cells (WBC), lymphocytes, platelets, platelet/lymphocyte ratio, or red cell distribution width (RDW) (*p* = 0.330, 0.384, 0.335, 0.149, 0.344, 0.06, and 0.892, respectively).

**Table 2:** Laboratory parameters by etiology.

Parameter	Renal agenesis (*n* = 158)	Multicystic dysplastic kidney (*n* = 37)	Nephrectomy (*n* = 27)	Atrophic kidney (*n* = 54)	Hypoplastic kidney (*n* = 120)	*p*
Albumin (g/dl)	4.1 (3.7–4.4)	3.8 (3.6–4.4)	4.5 (4.2–4.7)	4.4 (4.1–4.7)	4.4 (4.2–4.7)	<0.001
CRP (mg/dl)	4.2 (1.8–6.8)	3.3 (1.2–5.7)	3.2 (3–6.3)	3.6 (3.2–7.9)	3.3 (2.7–6.3)	0.330
CRP/albumin	1.02 (0.4–1.7)	0.86 (0.29–1.58)	0.75 (0.63–1.42)	0.87 (0.74–1.8)	0.76 (0.58–1.42)	0.384
WBC (10^3^/µl)	8.3 (6.49–11.69)	8.19 (6.12–10.44)	8.1 (6.34–10)	7.56 (6.02–9.38)	7.77 (6.48–10.08)	0.335
Lymphocytes (10^3^/µl)	2.58 (2–3.62)	2.5 (1.99–3.51)	3.1 (2.51–4.89)	2.76 (1.98–4.18)	2.84 (2.21–3.58)	0.149
Neutrophils (10^3^/µl)	4.61 (3.08–6.59)	4 (2.62–6.29)	3.75 (2.5–4.82)	3.22 (2.26–4.29)	3.55 (2.81–5.77)	0.001
Platelets (10^3^/µl)	324 (284–384)	318 (278–389)	295 (231–386)	333 (264–385)	327 (272–412)	0.344
NLR	1.68 (1.08–2.91)	1.43 (0.75–2.28)	1.08 (0.58–2.03)	1.12 (0.79–2.1)	1.22 (0.79–2.1)	0.002
PLR	129.2 (95–172.7)	125.8 (104.9–165.2)	109 (59–159)	115.5 (79.9–151.3)	120.8 (91–156.9)	0.06
RDW (%)	13.3 (12.6–14.5)	13.4 (12.6–14.3)	13.2 (12.5–14.6)	13.3 (12.9–14.7)	13.3 (12.5–14.6)	0.892

CRP, C-reactive protein; WBC, white blood cells; RDW, red cell distribution width; NLR, neutrophil-to-lymphocyte ratio; PLR, platelet-to-lymphocyte ratio.

A significant difference in median albumin levels was observed between the groups (*p* < 0.001). *Post-hoc* analyses showed that the multicystic dysplastic kidney group [3.8 (IQR 3.6–4.4) g/dl] had significantly lower albumin levels compared to the atrophic kidney [4.4 (IQR 4.1–4.7) g/dl, *p* = 0.04], nephrectomy [4.5 (IQR 4.2–4.7) g/dl, *p* = 0.014], and hypoplastic kidney [4.4 (IQR 4.2–4.7) g/dl, *p* < 0.01] groups. The renal agenesis group [4.1 (IQR 3.7–4.4) g/dl] also had significantly lower albumin levels compared to the atrophic kidney (*p* = 0.04), hypoplastic kidney (*p* < 0.001), and nephrectomy (*p* = 0.03) groups. No other group comparisons were statistically significant (adjusted *p* > 0.05).

A significant difference in median neutrophil levels was observed between the groups (*p* = 0.001). *Post-hoc* analyses showed that the renal agenesis group (4.61 [IQR 2–3.62] × 10^3^/µl) had significantly higher neutrophil levels compared to the atrophic kidney group (3.22 [IQR 2.26–4.29] × 10^3^/µl, *p* = 0.001). All other comparisons were not statistically significant (adjusted *p* > 0.05).

A significant difference in the neutrophil-to-lymphocyte ratio (NLR) was also observed (*p* = 0.002). The atrophic kidney group [1.12 (IQR 0.79–2.1)] had a significantly lower NLR compared to the renal agenesis group [1.68 (IQR 1.08–2.91), *p* = 0.004]. No other comparisons revealed statistically significant differences (adjusted *p* > 0.05).

The distribution of kidney size percentiles at the initial and final visits for the renal agenesis, multicystic dysplastic kidney, nephrectomy, atrophic kidney, and hypoplastic kidney groups is presented in Table 3.

**Table 3:** Kidney size percentiles at initial and final visit by etiology.

Visit period	Percentile group	Renal agenesis (*n* = 158)	Multicystic dysplastic kidney (*n* = 37)	Nephrectomy (*n* = 27)	Atrophic kidney (*n* = 54)	Hypoplastic kidney (*n* = 120)
Initial visit	<P2.5	8 (5.1%)	2 (5.4%)	1 (3.7%)	3 (5.6%)	9 (7.5%)
	P2.5–P9	6 (3.8%)	2 (5.4%)	0 (0%)	2 (3.7%)	5 (4.2%)
	P10–P24	7 (4.4%)	3 (8.1%)	3 (11.1%)	7 (13.0%)	3 (2.5%)
	P25–P49	11 (7.0%)	4 (10.8%)	2 (7.4%)	5 (9.3%)	8 (6.7%)
	P50–P74	30 (19.0%)	9 (24.3%)	4 (14.8%)	9 (16.7%)	19 (15.8%)
	P75–P89	26 (16.5%)	11 (29.7%)	2 (7.4%)	9 (16.7%)	32 (26.7%)
	P90–P97.5	25 (15.8%)	3 (8.1%)	7 (25.9%)	8 (14.8%)	16 (13.3%)
	>P97.5	45 (28.5%)	3 (8.1%)	8 (29.6%)	11 (20.4%)	28 (23.3%)
Final visit	<P2.5	0 (0%)	0 (0%)	0 (0%)	0 (0%)	3 (100%)
	P2.5–P9	4 (2.5%)	2 (5.4%)	2 (7.4%)	0 (0%)	3 (27.3%)
	P10–P24	3 (1.9%)	1 (2.7%)	2 (7.4%)	2 (3.7%)	5 (38.5%)
	P25–P49	13 (8.2%)	1 (2.7%)	1 (3.7%)	3 (5.6%)	19 (51.4%)
	P50–P74	25 (15.8%)	13 (35.1%)	2 (7.4%)	12 (22.2%)	25 (32.5%)
	P75–P89	21 (13.3%)	3 (8.1%)	3 (11.1%)	8 (14.8%)	18 (34.0%)
	P90–P97.5	28 (17.7%)	2 (5.4%)	5 (18.5%)	9 (16.7%)	24 (35.3%)
	>P97.5	64 (40.5%)	15 (40.5%)	12 (44.4%)	20 (37.0%)	23 (17.2%)

“P” refers to percentile (e.g., P2.5 = 2.5th percentile, P10 = 10th percentile).

## 4 Discussion

The findings from this study provide unique insights into the clinical outcomes and risk of kidney failure in pediatric patients with SFK of varying etiologies; notably, differences were observed in GFR, follow-up duration, and laboratory markers. While some of these findings are in agreement with the existing literature, others are in contradiction, highlighting the complexity of SFK and suggesting that it should be managed using individualized and unique management strategies.

The variation in GFR between patients with MCDK and renal agenesis observed in our study is supported by Matsell et al. (2) and Wakabayashi et al. (8), both of whom reported that MCDK patients tend to have decreased kidney function in the early stages. In't Woud et al. (1) also indicated that young children with MCDK may need close follow-up because their clinical course is distinctly different and they are at a higher risk for kidney injury. Some MCDK patients may retain their kidney function, challenging the widely accepted notion that kidney function always declines in these cases (9). This difference in findings highlights the need for patient-specific follow-up protocols for patients with different SFK etiologies (10).

One of the key reasons why patients with MCDK may show a greater decrease in GFR is the dysplastic nature of the renal parenchyma, which limits the compensatory capacity of the contralateral kidney compared to patients with renal agenesis. Hutchinson et al. (10) explained that in MCDK, there may be associated ipsilateral or contralateral abnormalities that further increase the risk of nephron loss. Matsell et al. (2) also emphasized that the structural immaturity of the contralateral kidney in MCDK patients predisposes them to an earlier decline in renal function.

While there were variations in the GFR values, the authors of this study observed no difference in the prevalence of hypertension and proteinuria in their patients, in contrast to the observations of Güngör et al. (11), who reported a higher incidence of these complications in patients with renal agenesis. Similar observations were reported by La Scola et al. (12), who indicated that such risk factors develop later in life, especially among children with SFK. Since hypertension and proteinuria are known to develop with age, the lack of significant variations in their levels observed in this study might be due to the shorter follow-up periods (7).

Our study revealed a common occurrence of compensatory hypertrophy among patients with renal agenesis, which is in agreement with the findings reported by McArdle et al. (5) and Westland et al. (6). While this type of compensatory alteration aids in the preservation of kidney function, it often indicates a possible risk for kidney injury, as shown in the studies by In't Woud et al. (4). Therefore, although hypertrophy is generally perceived as conferring a protective effect, it cannot and should not be considered the only indicator of the risk of developing CKD in the chronic phase.

Furthermore, laboratory findings revealed decreased albumin levels in MCDK cases and increased neutrophil counts in renal agenesis cases. The decreased albumin levels in MCDK patients may reflect early glomerular damage or increased renal permeability, even in the absence of overt proteinuria, as indicated by Wakabayashi et al. (8) and In’t Woud et al. (1). Moreover, albumin levels may act as a surrogate marker for overall nutritional and inflammatory status in these patients, with low levels being associated with an increased progression risk of CKD (1).

The elevated neutrophil counts observed in renal agenesis patients might suggest subclinical inflammation or compensatory immune response mechanisms. Matsell et al. (2) described that mild systemic inflammation is increasingly recognized in patients with congenital solitary functioning kidneys and may contribute to early endothelial dysfunction. This is echoed by In't Woud et al. (13), who cautioned that neutrophil elevation alone should not be interpreted in isolation but rather in conjunction with other renal stress or injury markers.

The studies by In't Woud et al. (1, 4) recommend that SFK patients be monitored closely through routine follow-up to minimize further renal injury. These observations suggest that patients who receive earlier follow-up are more severely affected; this was also seen in the studies by La Scola et al. (3) and Kolvek et al. (14), who emphasized the importance of early intervention in SFK patients.

Moreover, the lack of marked differences between the groups regarding proteinuria and hypertension indicates that these risk factors may be present in later years of life, as also documented by La Scola et al. (12). This remark highlights the need for continued follow-up, as patients with SFK might develop complications over time (4).

Overall, the results indicate that kidney size percentiles differ depending on the etiology, with notable differences observed between the multicystic dysplastic kidney, renal agenesis, nephrectomy, and hypoplastic kidney groups, in agreement with the literature, as highlighted by Marzuillo et al. (15), who emphasized the same. Marzuillo et al. (15) stressed that based kidney length percentiles do not apply to patients with obesity, which is the case with the hypoplastic kidney group, where an asymmetric distribution was seen. The simultaneous existence of low and high percentiles in the hypoplastic group indicates the presence of both causes and factors associated with the general somatic development and body composition of the patients. The normative percentile study by Obrycki et al. (16) also concurs with the suggestion that the positive shifts in percentiles seen in the multicystic dysplastic kidney group represent the normal trends, and they are likely to be associated with the functional load distribution in these kidneys.

According to the normative ultrasound study by Calle-Toro et al. (17), the increasing percentiles in the multicystic dysplastic kidney group indicate positive development. The increase in the higher percentiles seen in the renal agenesis and nephrectomy subgroups is typical of compensatory enlargement of the remaining functional kidney. Overall, these conclusions highlight the importance of the causes of disease, growth patterns, and environmental factors in relation to kidney size percentiles, together with highlighting the need for correct evaluation techniques to avoid misinterpretation (15, 16).

In summary, the study highlights that SFK in childhood can have a variety of clinical courses, underscoring the requirement of individualized follow-up and management strategies. As noted by La Scola et al. (12) and Marzuillo et al. (7), long-term and multidisciplinary care of this patient group is essential to achieving the best outcomes. By establishing striking departures in GFR values, laboratory variables, and other risk factors for kidney injury, this research extends the comprehension of the long-term exposures related to various SFK etiologies (1, 5, 8). The findings from this study also reinforce the need for individualized treatment plans coupled with regular follow-up to lessen the effects of hypertension, proteinuria, and CKD in SFK patients.

This study provides a detailed overview of clinical features and kidney function in the pediatric population with SFK due to renal agenesis, MCDK, nephrectomy, atrophic kidney, and hypoplastic kidney. The findings indicate significant differences in GFR, follow-up duration, and laboratory markers between the groups, making them understand the long-term risks to which these patients are predisposed and how best to manage them.

The large variations in baseline and final GFR between the groups, particularly the lower GFR observed in patients with MCDK, stress the elevated risk of developing kidney function loss in this subset of patients. These findings also underscore the necessity to regularly monitor MCDK patients to reduce the chances of CKD development. The higher annual rate of GFR decline observed in some groups calls for specific follow-up schedules related to the underlying causes of the condition.

Although there were discrepancies in the occurrence of GFR, hypertension, and proteinuria, there were no statistically significant differences between the comparisons, which implies that these risk factors may arise late in the disease course and need continuous surveillance. In addition, there were differences in laboratory parameters: MCDK patients had lower albumin levels, while renal agenesis patients had higher neutrophil levels. These results imply that these laboratory tests can be useful in diagnosing renal injury in the early stages of the disease and its management.

Finally, the assessment of kidney volume growth percentiles showed that the loss of one kidney is mostly compensated by hypertrophy of the remaining kidney, especially in cases of renal agenesis. However, such compensatory growth does not automatically eliminate the risk of kidney function deterioration, hence emphasizing the need for close and tailored follow-up of SFK patients.

To summarize, this research extends previous recommendations for SFK management to include chronic renal failure in multicystic dysplastic kidneys and renal agenesis in children. Specifically, the more pronounced GFR decline observed in MCDK patients may be due to both structural deficits and hidden bilateral anomalies (2, 10). Decreased albumin levels and increased neutrophil levels provide a biochemical signal of subclinical damage, and their role as early markers of kidney injury should be investigated further in future prospective studies (1, 8). In cases of multicystic dysplastic kidney, early diagnosis, consistent follow-up, and appropriate management help in averting chances of CKD and other associated risks. Additional studies are needed to establish optimal management strategies for specific mechanisms of kidney injury in different types of SFK.

## Data Availability

The raw data supporting the conclusions of this article will be made available by the authors without undue reservation.
